# Supplementary material for: In Vitro Metabolism of Donepezil in Liver Microsomes Using Non-Targeted Metabolomics
Source: Pharmaceutics. 2021 Jun 23;13(7):936. doi: 10.3390/pharmaceutics13070936 (PMC8309179; doi:10.3390/pharmaceutics13070936)
Supplement: Supplementary file 1 [file pharmaceutics-13-00936-s001.zip › pharmaceutics-1250726-supplementary.pdf]

pharmaceutics

Supplementary Materials: In Vitro Metabolism of Donepezil in Liver Microsomes Using Non-Targeted Metabolomics

Sin-Eun Kim, Hyung-Ju Seo, Yeojin Jeong, Gyung-Min Lee, Seung-Bae Ji, So-Young Park, Zhexue Wu, Sangkyu Lee, Sunghwan Kim and Kwang-Hyeon Liu

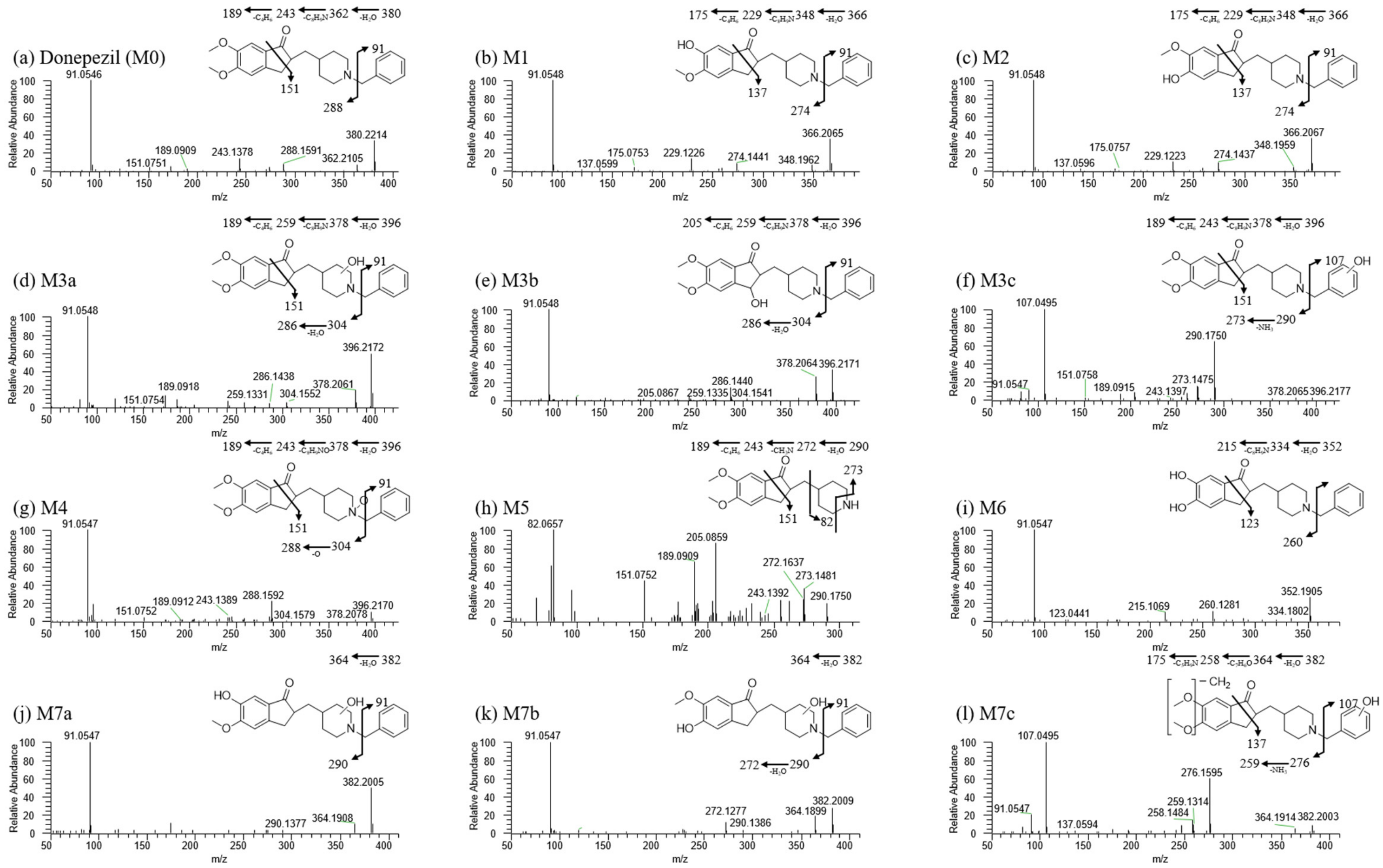

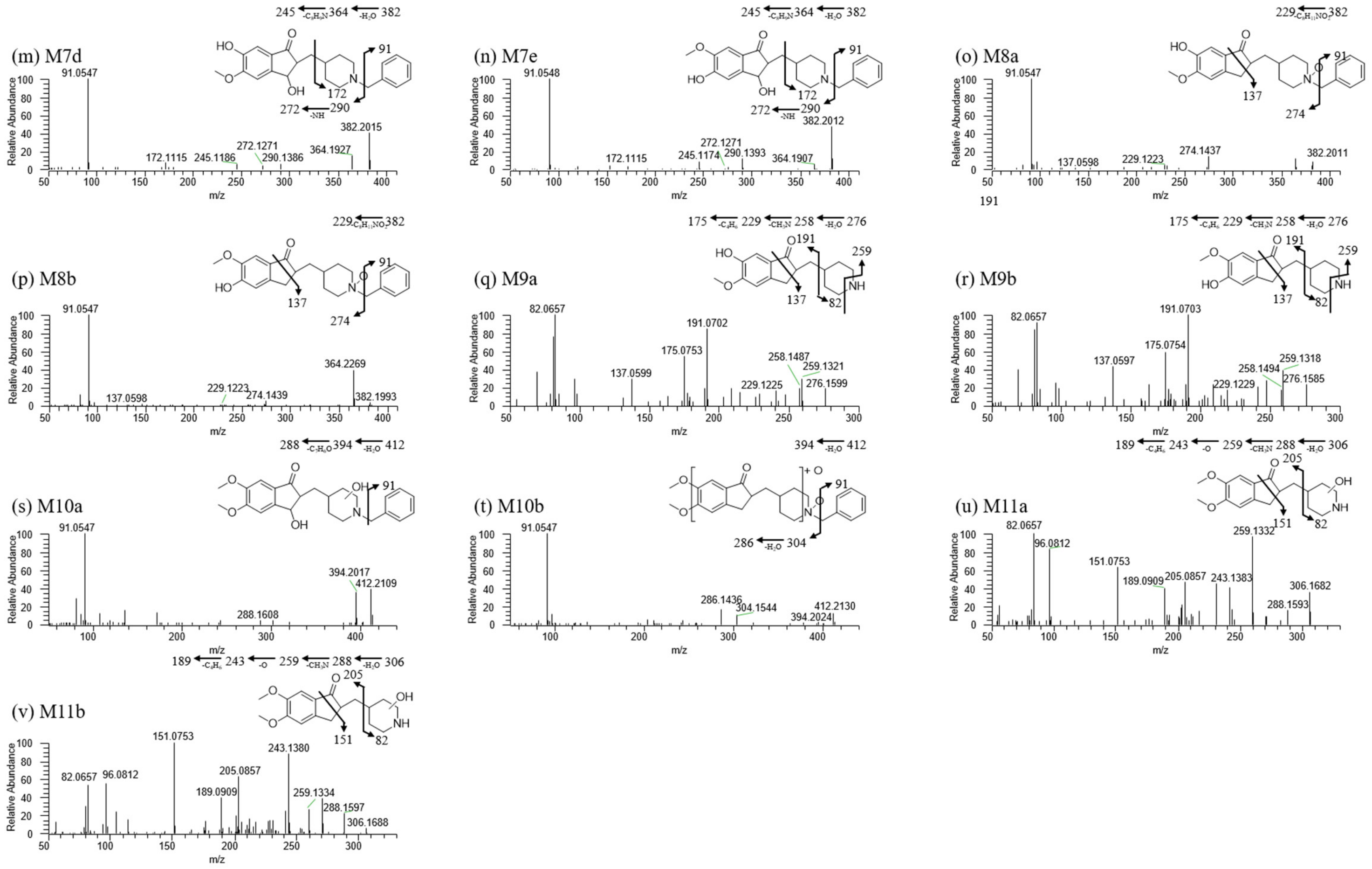

Figure S1. (**a**) Representative product ion scan mass spectra of donepezil (**M0**) and (**b–v**) metabolites of donepezil (**M1–M11**), and their proposed fragmentation scheme. The information on each metabolite is given in Table 1.

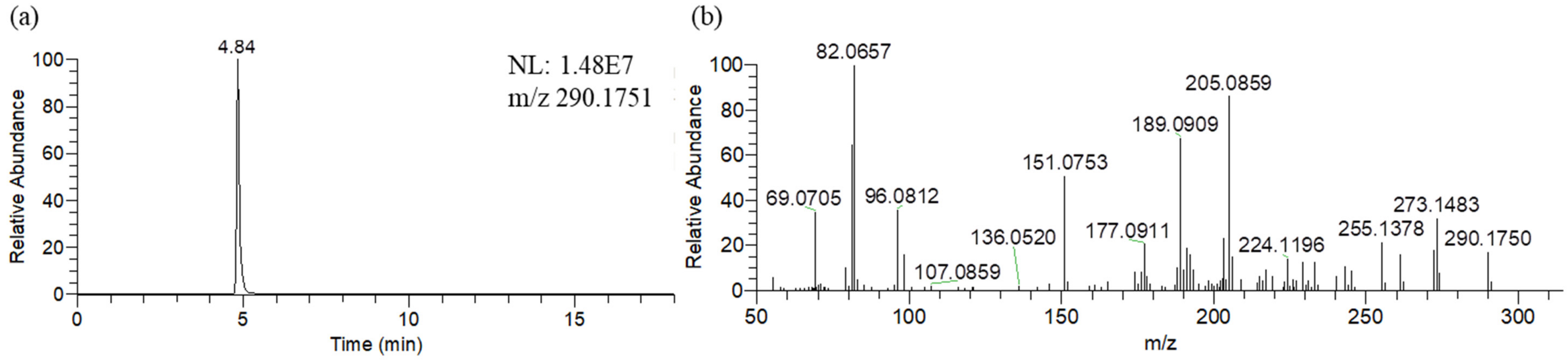

Figure S2. (**a**) Representative extracted ion chromatograms and (**b**) product ion scan mass spectrum of *N*-desbenzyldonepezil (**M5**) authentic standard.
